# Characterization of bacterial communities associated with seabed sediments in offshore and nearshore sites to improve Microbiologically Influenced Corrosion mitigation on marine infrastructures

**DOI:** 10.1371/journal.pone.0309971

**Published:** 2024-09-04

**Authors:** Daniele Ghezzi, Gianmarco Mangiaterra, Arianna Scardino, Mauro Fehervari, Mauro Magnani, Barbara Citterio, Emanuela Frangipani

**Affiliations:** 1 Department of Biomolecular Sciences, University of Urbino Carlo Bo, Urbino (PU), Italy; 2 R&D Engineering, Asset Based Services—Saipem SpA, Fano (PU), Italy; The University of Akron, UNITED STATES OF AMERICA

## Abstract

Microbiologically Influenced Corrosion (MIC) is one of the main threats for marine infrastructures, leading to severe safety and environmental risks associated with structural failures and/or leakages of dangerous fluids, together with potential huge economic losses and reputational damage for the involved parts. For a safe design and a proper installation of infrastructure systems in contact with the seabed, a deep knowledge of the site-specific microbial community of the sediments should be beneficial. Therefore, in addition to the simple detection or the sole quantification of Sulphate-Reducing Bacteria (SRB), the whole characterization of the microbial members involved in MIC phenomena is desirable. In this study, 16S rRNA-based comparison between bacterial communities thriving in offshore and nearshore marine sediments was performed, with a focus on the main bacterial groups putatively responsible for MIC. The nearshore sediments were significantly enriched in bacterial members associated with human and organic compounds contamination belonging to the *Bacteroidota*, *Desulfobacterota*, and *Firmicutes* phyla, while the offshore sediments hosted *Alphaproteobacteria*, *Nitrospinota*, and *Nitrospirota* members, representative of a low anthropogenic impact. Quantitative PCR targeting the *dsrA* gene and detailed community analyses revealed that the nearshore sediments were significantly enriched in SRB mainly affiliated to the *Desulfobulbus* and *Desulfosarcina* genera potentially involved in biocorrosion, compared to the offshore ones. These results suggest that the bacterial community associated with the high concentration of organic compounds derived by an elevated anthropogenic impact is likely to favour MIC. Such observations highlight the importance of microbiological investigations as prevention strategy against MIC processes, aiming both at characterizing sites for the establishment of new infrastructures and at monitoring those already installed.

## Introduction

Corrosion of metal infrastructures in aquatic settings raises significant worries regarding environmental pollution, potential harm to aquatic ecosystems, degradation of infrastructure integrity, and substantial economic losses [1]. Metal abiotic deterioration is further accelerated by the involvement of biofouling and Microbiologically Influenced Corrosion (MIC), two mechanisms mediated by the biological activity of different organisms [2]. Biofouling refers to the accumulation and growth of various micro- and macro- organisms, including plants and/or animals (*e*.*g*., algae, mussels, bernacles) on a specific surface [3], while MIC has been defined by the National Association of Corrosion Engineers (NACE) and the American Society for Testing and Materials (ASTM) as “*corrosion affected by the presence or activity*, *or both*, *of microorganisms*” (ASTM G193 2022) and denotes a complex process caused only by microorganisms and/or their metabolites on metallic surfaces that can accelerate corrosion by 10–1,000 times [4]. Although the potential for MIC can be found in both freshwater and seawater, the latter represents an extremely harsh corrosive environment, favoring MIC and leading to the breakdown or degradation of metallic materials also through an electrochemical reaction stimulated by the naturally high salt concentration, combined with its elevated electrical conductivity [5, 6]. Indeed, the high sulfate content of seawater (*i*.*e*., 2.5–5 g L^-1^) [7] also supports the metabolic activity of specific bacterial taxa responsible for MIC, thus indirectly boosting the corrosive process.

MIC constitutes the main significant challenge for the maintenance of marine infrastructures, deeply affecting many industrial fields, including the oil, gas and water utilities ones [8], being the direct cause of catastrophic corrosion failures with very high associated damage costs. In this regard, according to estimates by NACE International, the total cost of corrosion is *ca*. US$ 2.5 trillion/year globally, with MIC accounting for 20%–40% [9–11]. Furthermore, MIC and abiotic corrosion often occur simultaneously, making it challenging to differentiate the costs attributed to these two processes independently [12]. The key categories of bacteria linked to metal corrosion vary according to their metabolic activities, primarily encompassing Sulfate-Reducing Bacteria (SRB), Acid Producing Bacteria (APB), Sulfur-Oxidizing Bacteria (SOB), Metal-Oxidizing Bacteria (MOB), Metal-Reducing Bacteria (MRB), Slime-Forming Bacteria (SFB) and microorganisms that produce organic acids and are able to grow as biofilms [4, 13]. Usually, aerobic and facultative microorganisms tend to be found in the outer layers of the biofilm and establish the conditions that promote metal corrosion, but most corrosion has been attributed to anaerobes that are located in the inner layer of the biofilm [14]. Among these, SRB are the most reported corrosion-related microbes, as well as the best characterized. SRB play key roles in the carbon and sulfur cycles in marine environments by participating in the dissimilatory sulfate reduction process [15, 16]. They are estimated to perform half of the organic matter mineralization and degrade a variety of byproducts [17]. As a consequence of the organic matter degradation and sulfate reduction, SRB produce hydrogen sulfide (H_2_S) that accumulates in sediment and diffuses towards the shallower sea layers where it promotes corrosive processes to the marine infrastructures [18]. Indeed, SRB are considered one of the main types of microorganisms responsible for MIC and can cause severe issues both in terms of hydrocarbon contaminants pollution and economic losses, causing corrosive phenomena in downstream processes of companies working on marine infrastructures and offshore oil producers [19]. Moreover, SRB are able to adhere to metal surfaces and form biofilms characterised by the presence of a thick exopolysaccharide (EPS) matrix, that contributes to the establishment of a localized, oxygen-depleted, and corrosive microenvironment. The H_2_S produced by SRB reacts with the metal surface, leading to the formation of iron sulfides (FeS) that can initiate pitting and under deposit corrosion. These localized areas of corrosion can, in turn, lead to structural damages and the eventual failure of metal components [20].

To prevent and limit MIC in marine infrastructures, multiple strategies are currently in use aiming at fighting the adhesion and biofilm formation of the microorganisms on the surfaces. The most applied approach includes the employment of chemicals before and/or during the usage of the infrastructures, which are highly effective, but also toxic for non corrosion-causing organisms present in the marine ecosystem, causing great environmental concern in the long run [13]. Additional strategies include the development of coatings appliable on the surfaces with anti-adhesion and antibiofilm capacity, but their efficacy over time is often limited [21, 22], and the cathodic protection, which is useful to reduce the migration of electron during corrosion, can occasionally promote bacterial corrosion [13]. Moreover, applicable technical standards are duly implemented during design to properly consider all known aspects of corrosion [23, 24], since operating practices may create conditions that promote the proliferation and spread of biocorrosive microorganisms, especially in offshore oil-producing platforms [25]. Moreover, in specific sites where the SRB presence and H_2_S abundance have been definitely recognized in the sediments (*e*.*g*., Black sea and Baltic sea), dedicated analyses are adopted to select materials and design cathodic protection of strategic infrastructures which pass through such critical environments, like large export gas pipelines [26, 27].

MIC assessment in the oil and gas industry is mainly estimated using culture-based methods (*i*.*e*., Most Probable Number determination), which are biased by the complex growth conditions of SRB. Culture-independent molecular studies on bacterial communities impacting MIC are listed by NACE as non mandatory methods, although there has been much more emphasis in the past decade on the potential use of these molecular methods to aid in defining the potential risk for MIC and provide insight into microbial control strategy to list sites that requires periodic monitoring [28, 29].

Apart from these strategies, which are employed regardless of the microbiome composition of the sites, there are currently no additional standard mandatory analyses planned prior to the installation of marine infrastructures, to detect the presence of specific microorganisms potentially capable of carrying out corrosion in the sites of interest.

In this work, the microbial communities associated to anthropogenically-impacted nearshore and to less impacted offshore marine sediments were analysed through metabarcoding sequencing targeting the V3 and V4 hypervariable regions of the 16S rRNA gene to gain information on the possible proneness of these two different sites to develop MIC, affecting marine infrastructures. Furthermore, the quantification of SRB was assessed through quantitative PCR targeting the *dsrA* gene involved in the dissimilatory sulfate reduction process. Sediment samples were collected from the Trieste port and the Norwegian Barents Sea in the proximity of an oil/gas extraction platform. Trieste port is part of the contaminated Sites of National Interest, which include several areas featured by high levels of hazardous contaminants that can provoke serious damages to the environment, to the human health, and to the cultural heritage [30–32]. Indeed, ports suffer numerous dangerous environmental impacts mainly due to the discharge of petroleum-based pollutants in combination with the morphological structure of the port area, which is bordered by lands and promotes the accumulation of contaminants on site that require a complex remediation policy [33]. Conversely, the Barents Sea is an open marine system, where large amounts of dispersed organic matter from the northern Atlantic and Arctic oceans interchange, mainly precipitating on the sea bottom [34–36]. The sampling site in the Norwegian Barents Sea is featured by a different human-dependent contamination typology, which is represented by the presence of a drilling platform installed at 100 km far from the coast in the nearby of the collected sediments. These samples have been chosen as case studies to assess the microbial community composition of two differentially human-impacted locations, to evaluate the ecological health of marine sediments and to discuss the importance of monitoring the evolution of microbial groups involved in corrosion processes, as a strategy to prevent and/or mitigate MIC.

## Materials and methods

### Sampling and environmental parameters

**Fig 1:**
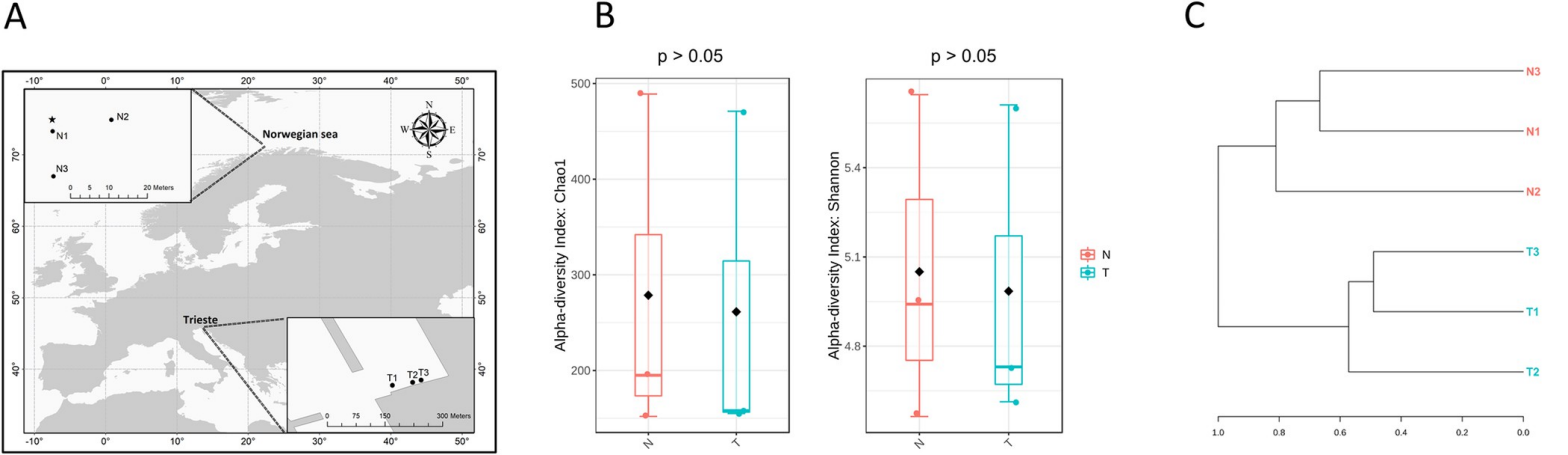
Samples’ geographical localization and microbial diversity. A) Geographical map of Norway (N) and Trieste (T) sample sites. The star indicates the position of the Scarabeo 8 offshore semisubmersible drilling unit. B) Chao1 and Shannon indices of microbial communities at Amplicon Sequence Variant (ASV) level detected in N and T sediments. C) Dendrogram of N and T microbial communities at ASV level.

Sediments (between 100 and 200 g each) were collected in March 2021, during routine operations conducted by Saipem S.p.A., and stored in sterile containers at 4°C during transport to the laboratory, where they were kept at -80°C until processed. For each sediment sample, three DNA extractions were performed and pooled. Norway sediments (N1, N2, and N3) were collected from the surface of the seabed in undisturbed locations in the Goliat field at 370 m depth in the proximity of the Saipem’s Scarabeo 8 offshore semisubmersible drilling rig (Fig 1A, S1 Table). N sediments were collected with a Van Veen grab sampler at 4 m South, 15 m East, and 15 m South of the platform, respectively (S1 Table, Fig 1A). Trieste (Italy) sediments (T1, T2, and T3) were collected from a seabed close to a Saipem SpA base within a 50-m port area at 8–9 m depth.

Environmental parameters were either measured in the lab upon sample reception (pH) or retrieved from dedicated databases and Saipem S.p.A. internal reports, as detailed in S2 Table. Since this work focuses on the environmental features potentiating MIC, the material of the infrastructures and the activity of the microorganisms on them were not investigated.

### Total DNA extraction, 16S rRNA gene amplification and sequencing

Total DNA was extracted from around 250 mg of homogenized sample using the PowerSoil DNA Isolation Kit (Qiagen) with slight modifications, as previously described [37]. Three independent extractions were performed for each sample, and the obtained DNA was pooled. The V3 and V4 hypervariable regions of the 16S rRNA gene were amplified using 341F (5’-CCTACGGGNGGCWGCAG-3’) and 806R (5’-GACTACHVGGGTATCTAATCC-3’) primers pair [38] with 5’-Illumina adapters and for MiSeq Illumina sequencing. The amplifications were performed in 25 μl final volume containing 1x KAPA HiFi HotStart Ready Mix, 0.2 μM each primer, and 30 ng DNA (Bio-Fab Research s.r.l., Rome—Italy). The amplification program included a first DNA denaturation at 95°C for 3 minutes, followed by 25 cycles of denaturation at 95°C for 30 seconds, primer annealing at 55°C for 30 seconds, extension at 72°C for 30 seconds, and a final extension at 72°C for 5 minutes. Amplicons were submitted to the library preparation and Illumina MiSeq sequencing platform for indexing and pair-end sequencing (2 × 300 bp; reagent kit, v2) at the sequencing service Bio-Fab Research s.r.l.

The Illumina sequencing raw data were deposited in the Sequence Read Archive of NCBI under accession number PRJNA1053306.

### Data processing

The output reads were first trimmed for their adapters and primer sequences and then checked for chimera and quality by using QIIME2 software version 2022.2.0. Reads were processed into Amplicon Sequence Variants (ASVs) using the DADA2 package version 1.14, as described in [39]. Statistical analyses, rarefaction curves, alpha and beta diversity indices calculation were performed by using Microbiome Analyst software [40] and Primer-E version 7 (Primer-E Ltd, Plymouth, UK). The taxonomic assignment of the resulting ASVs was performed by querying the ASVs against SILVA SSU database version r138.1 [41]. Eukaryotic, mitochondrial, and chloroplast sequences were removed from further analyses. Differentially abundant taxa were assessed by performing ANOVA-based single-factor analysis in Microbiome Analyst.

An unrooted phylogenetic tree was built with the neighbor-joining method, maximizing the likelihood with a gamma model distribution, and using the most abundant ASVs representative of each sample together with the most closely related sequences retrieved from the NCBI database (Best Blast Hits). MEGA11 [42] was used to construct phylogenetic trees based on ClustalW sequences alignment and neighbor-joining clustering method with 1,000 non-parametric bootstrap replicates (model: Jukes-Cantor; rates among site: uniform rates; gap/missing data treatment: pairwise deletion). iTOL was used to visualize the phylogenetic tree.

### Quantitative PCR targeting the drsA gene

The SRB population was quantified in the sediment samples by qPCR assays targeting the alpha subunit of the dissimilatory sulfate reductase (*dsrA*) gene. The reactions were performed using 10 μl of the 2x QuantiNova SYBR Green PCR Master Mix (Qiagen), 2 μl of template DNA and 0.2 μM of each primer of the previously described pair DSR1-F+ (5’-ACSCACTGGAAGCACGGCGG-3’)/DSR-R (5’-GTGGMRCCGTGCAKRTTGG-3’) [43].

A calibration curve was built using 10-fold dilutions of the purified *dsrA* amplicon from the genomic DNA of *Desulfovibrio vulgaris* ATCC 29579, according to a previously validated qPCR setting [44], properly modified for this target gene. The gene copy number was determined dividing the total quantified DNA by the weight of one amplicon copy, *i*.*e*., 2.42 × 10^− 10^ ng, calculated considering the amplicon size (221 bp) and the weight of 1 bp (1.10 × 10^− 12^ ng). Each sample was tested in duplicate in two independent reactions. The results were expressed as gene copy number per gram of sediment.

## Results

### Sequencing results and diversity analysis

A total of 54,771 reads were obtained of which 28,865 from the Norway (N) samples and 25,906 from the Trieste (T) samples (S1 Table). The processed ASVs were 836 and 784 for N and T microbial communities, respectively, with N2 and T2 having the highest number of ASVs (*i*.*e*., 489 and 471, respectively). The rarefaction curves showed that the sequencing provided sufficient depth to investigate the microbial community composition and diversity (S1 Fig). The alpha diversity indices calculation revealed that Chao1 and Shannon mean values were not significantly different between N and T sediments (Fig 1B), whereas Bray-Curtis analysis at ASV level revealed two clusters corresponding to the two different sample groups, with T communities being more similar among each other compared to N ones (Fig 1C).

### Taxonomy composition of the microbial communities in offshore and nearshore sediments

**Fig 2:**
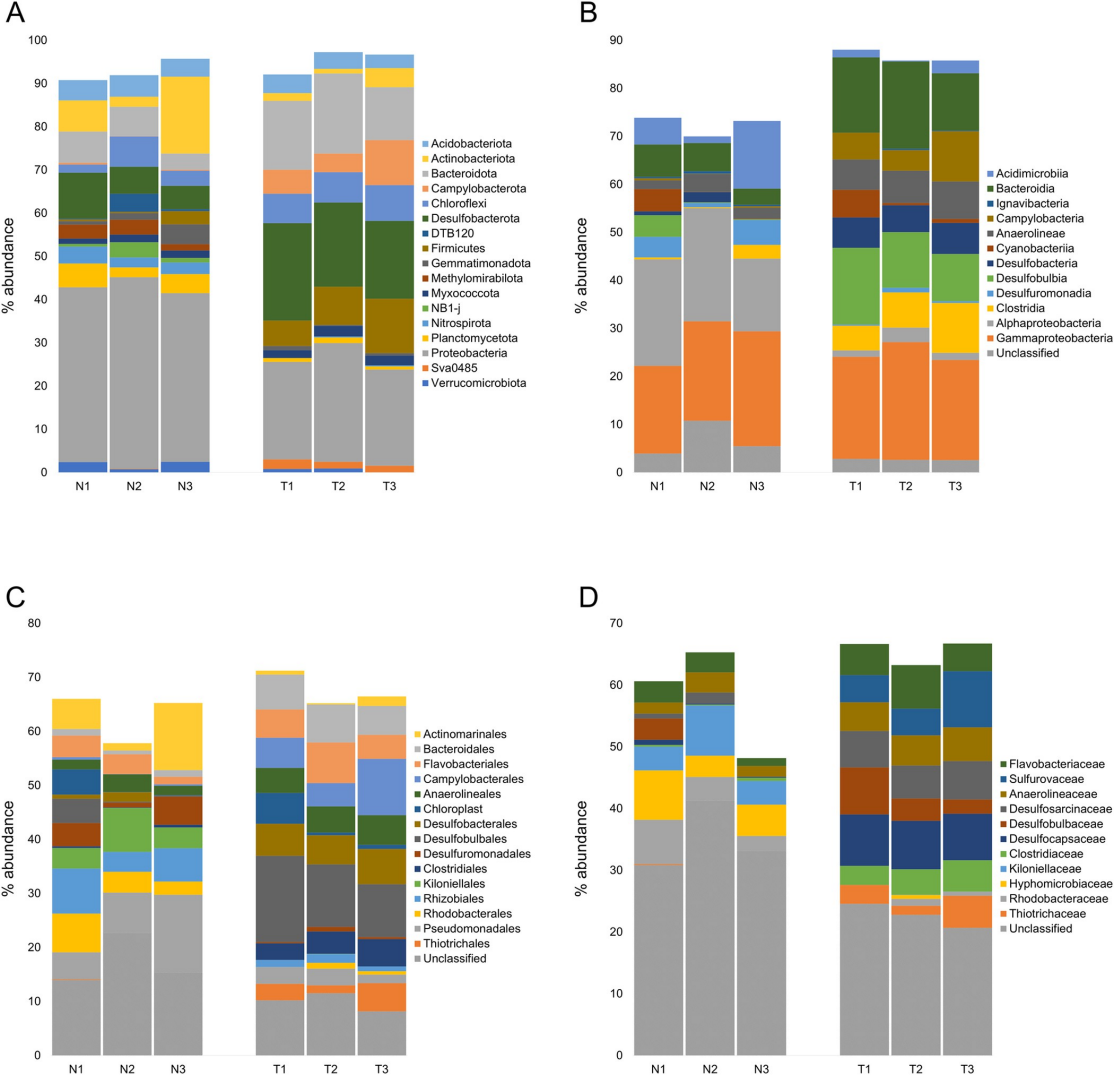
Microbial community composition. Taxonomy composition of offshore N and nearshore T microbial communities at A) phylum level, with abundances >2% in at least one sample, B) class, C) order, and D) family levels, with abundances >5% in at least one sample.

The offshore N sediments presented 14 high abundant (>2% in at least one sample) bacterial phyla, while nearshore T sediments were composed of 10 high abundant bacterial phyla. Among these, DTB120, *Gemmatimonadota*, *Methylomirabilota*, NB1-j, *Nitrospirota*, *Planctomycetota*, and *Verrucomicrobiota* were more abundant in N, while *Campylobacterota*, *Myxococcota*, and Sva0485 prevailed in T (Fig 2A). Proteobacteria represented the most abundant phylum in both sites and was mainly composed of *Alphaproteobacteria* and *Gammaproteobacteria* classes. The average abundance (avg.) of *Gammaproteobacteria* accounted for 23% in T and 21% in N, while *Alphaproteobacteria* were highly abundant in N only (avg. 21%), and poorly present in T (avg. 2%) (Fig 2B). *Gammaproteobacteria* were distinguished between N and T at lower taxonomic levels. In N, they were taxonomically affiliated to genera of the *Pseudomonadales* order, *i*.*e*., *Alcanivorax*, *Amphritea*, and C1-B045 of the *Porticoccaceae* family, and to genera of the *Steroidobacterales* order, *e*.*g*., *Woeseia*. On the other hand, *Gammaproteobacteria* in T were mainly composed of unclassified genera belonging to *Thiotrichaceae*, *Thiomicrospiraceae*, and *Sedimenticolaceae* families (Fig 2 and S2 Fig). The most abundant families of *Alphaproteobacteria* in N included *Rhodobacteraceae*, *Hyphomicrobiaceae*, *Kiloniellaceae*, and unclassified *Defluviicoccales* (Fig 2 and S2 Fig). The second most abundant phylum was represented by *Actinobacteriota* in N (avg. 9%; 2% in T) and *Desulfobacterota* in T (avg. 20%; 7% in N). *Actinobacteriota* in both sites were mainly affiliated to unclassified *Actinomarinales* order (Fig 2 and S2 Fig). *Desulfobacterota* in T were composed of the *Desulfobacterales* and *Desulfobulbales* orders and *Desulfosarcina* and *Desulfobulbus* genera, whereas in N they were affiliated to the *Desulfuromonadales* order and *Geopsychrobacter* genus (Fig 2 and S2 Fig).

*Bacteroidota* and *Firmicutes* phyla accounted for 16% (avg.) and 9% in T, whereas in N they composed 6% and 1% of the community, respectively. In T, *Bacteroidota* were composed of *Cyclobacteriaceae* and *Flavobacteriaceae* families, whereas *Firmicutes* were composed of *Erysipelotrichaceae* (mainly *Turicibacter*), *Clostridiaceae* (mainly *Clostridium*), and *Peptostreptococcaceae* (mainly *Romboutsia*). *Campylobacterota* were also highly abundant in T only (avg. 7%) and were composed of *Sulfurimonadaceae* (mainly *Sulfurimonas*) and *Sulfurovaceae* (mainly *Sulfurovum*) families (S2 Fig).

Additional phyla present in N and T were *Planctomycetota* (avg. 4% and 1%, respectively), *Nitrospirota* (avg. 3% and <1%), *Acidobacteriota* (avg. 5% and 4%), *Myxococcota* (avg. 2% in both sites), *Chloroflexi* (avg. 4% and 7%), *Verrucomicrobiota* (avg. 2% and <1%), *Gemmatimonadota* (avg. 2% and <1%), and Sva0485 (avg. <1% and 2%) (Fig 2).

Abundant (>3% in at least one sample) classified genera belonging to these phyla included *Lutibacter* of the *Flavobacteriaceae* family (*Bacteroidota*), *Nitrospira* (*Nitrospirota*), and the acidobacterial Subgroup 10 and Subgroup 23 genera of the *Thermoanaerobaculaceae* family. Whilst all the abundant phyla in T were also present in N, some abundant phyla in N were not present in T, *i*.*e*., DTB120 (avg. 2%), NB1-j (2%), and *Methylomirabilota* (3%) (S2 Fig).

**Fig 3:**
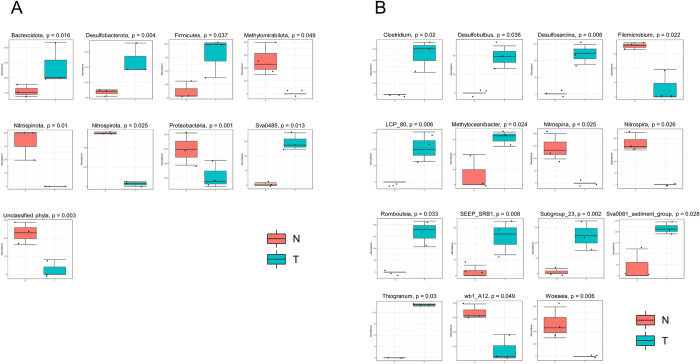
Microbial taxa enriched in Trieste and Norway sediments. Differentially abundant microbial phyla (A) and genera (B) between offshore N and nearshore T sediments. Only taxa significantly (p<0.05) enriched in N or T are shown.

Differentially abundant taxonomic analysis was performed to compare the microbial communities of the two different location and identify the bacterial taxa significantly describing each sampling location. At phylum level, T sediments were significantly (p<0.05) enriched in *Bacteroidota*, *Desulfobacterota*, *Firmicutes*, and Sva0485 (Fig 3A). Their affiliated genera significantly present in T were *Clostridium*, *Desulfobulbus*, and *Desulfosarcina* (Fig 3B). On the other hand, N sediments were featured with the presence of *Methylomirabilota*, *Nitrospinota*, and *Nitrospirota* phyla, along with members of *Proteobacteria* (mainly of the *Alphaproteobacteria* class). At genus level, N were characterized by *Nitrospina*, *Nitrospira*, wb1_A12, and *Woeseia* (Fig 3B). Taxonomically unclassified sequences at phylum level were significantly abundant in N sediments (Fig 3A).

### Phylogenetic analysis of the most abundant ASVs identified in offshore and nearshore sediments

**Fig 4:**
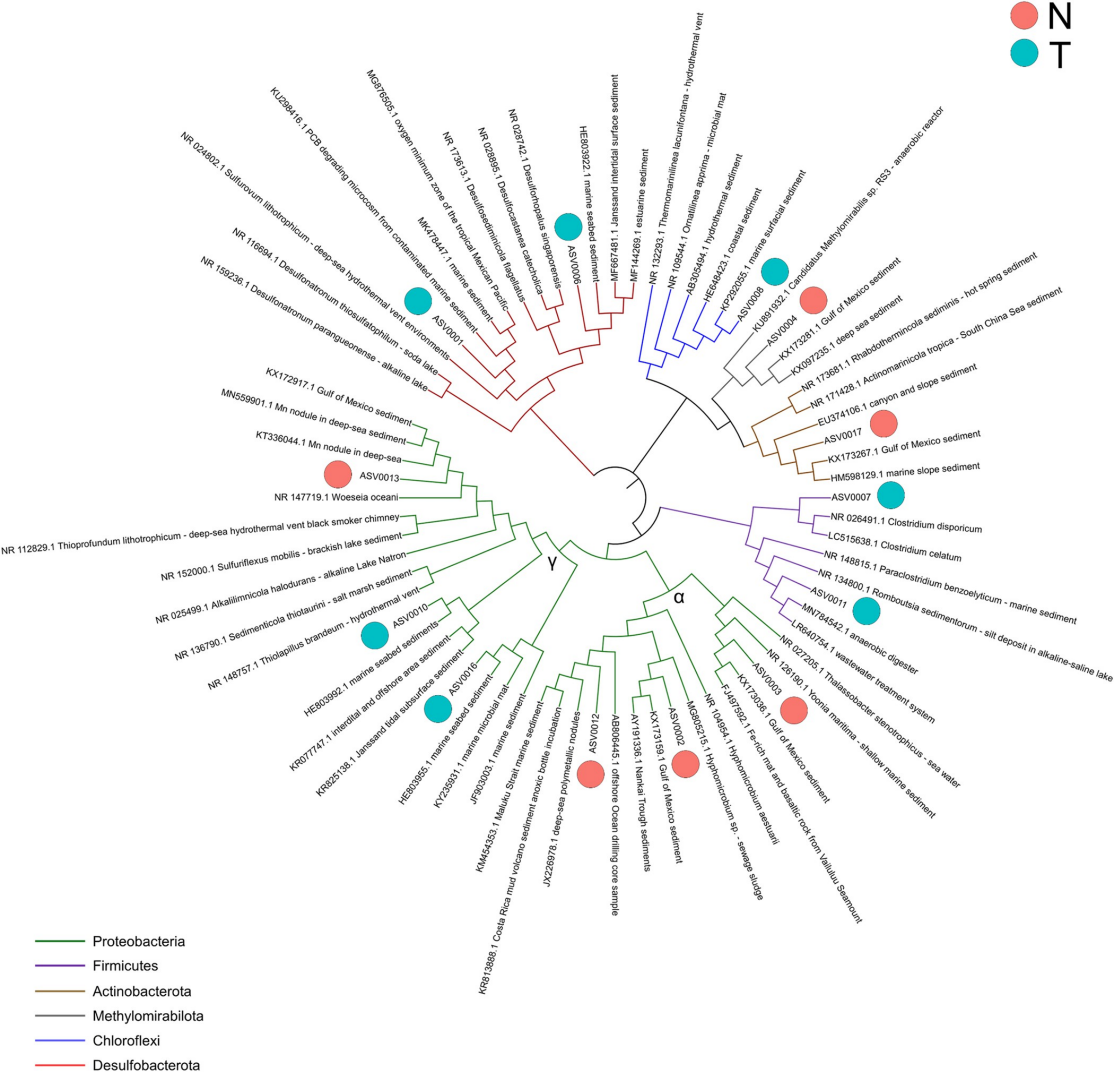
Phylogeny of ASVs identified from Trieste and Norway sediments. Phylogenetic tree showing the most abundant ASVs identified from offshore N and nearshore T microbial communities with their most similar sequences retrieved from the NCBI database. The ASVs characterizing N and T sediments are indicated with a red and blue dot, respectively.

The most abundant ASVs obtained from both N and T samples analyses were used to build a phylogenetic tree together with their closest cultured and uncultured relatives retrieved from the NCBI database. These ASVs were taxonomically affiliated to the dominant phyla identified in the taxonomic analysis, *i*.*e*., *Proteobacteria*, *Firmicutes*, *Actinobacterota*, *Methylomirabilota*, *Chloroflexi*, and *Desulfobacterota* (Fig 4).

*Methylomirabilota*- and *Actinobacteriota*-affiliated ASV0004 and ASV0017, respectively, were enriched in N sediments and shared >99% with uncultured representatives retrieved from non-contaminated sediments collected from an area within the Gulf of Mexico, where in 2010 the largest oil spill at sea in history took place after the Deepwater Horizon drilling platform explosion [45, 46]. ASV0004 shared low sequence identity (88%) with an isolate belonging to Candidatus *Methylomirabilis* retrieved from a reactor with anaerobic oxidation of methane coupled with nitrite reduction, while ASV0017 shared ~88% with a strain of *Rhabdothermincola sediminis* isolated from hot spring sediments. ASV0013 abundant in N shared ~96% with a strain of *Woesia oceani* isolated from coastal sediments and >99% with uncultured bacteria retrieved from non-contaminated sediments of the Gulf of Mexico and from manganese nodules in deep sea sediments. Three additional ASVs characterizing N sediments were affiliated with the *Alphaproteobacteria* class: ASV0002, which was >99% similar to clones retrieved from non-contaminated sediments of the Gulf of Mexico and shared ~97% identity with an isolate of the *Hypomicrobium aestuarii* species; ASV0003, which shared high nucleotide identity with an uncultured representative retrieved from an oil-contaminated surface brown layer of the Gulf of Mexico and shared ~97% with bacterial isolates of *Yoonia maritima* and *Thalassobacter stenotrophicus* species; ASV0012, which was >99% similar to clones retrieved from deep sea metallic nodules and offshore drilling samples and shared ~97% identity with a *Hypomicrobium aestuarii* strain (Fig 4).

Among the seven ASVs characterizing T sediments, two were affiliated to *Desulfobacterota*, *i*.*e*., ASV0001 and ASV0006. ASV0001 shared ~99% nucleotide identity with a cultured bacterial strain of *Sulfurovum lithotrophicum* isolated from deep-sea hydrothermal vents and with uncultured bacteria retrieved from contaminated marine sediments, whereas ASV0006 shared 94–95% identity with cultured strains of *Desulfosediminicola*, *Desulfocastanea*, and *Desulforhopalus* genera but formed a separated branch in the phylogenetic tree with uncultured clones from marine seabed sediments including the Janssand intertidal surface sediment (Fig 4). Additionally, the gammaproteobacterial ASV0010 and ASV0016 shared high percentage (>99%) with uncultured clones retrieved from marine sediments, including the Janssand ones (ASV0010 only), and low percentage (<93%) with cultured bacteria deposited in NCBI. ASV0010 shared ~92% with a cultured strain of *Thioprofundum lithotrophicum* isolated from a hydrothermal vent, whereas ASV0016 shared ~91% with a cultured strain of *Sedimenticola thiotaurini* isolated from a salt marsh sediment. One additional ASV characterizing T sediments was ASV0008, which shared >99% identity with uncultured clones retrieved hydrothermal and coastal sediments, and ~89% with a strain of *Thermomarinilinea lacunifontana* (belonging to the *Chloroflexi* phylum) isolated from a hydrothermal vent. Two *Firmicutes*-affiliated ASVs are indicators of anthropogenic impact of Trieste sediments, *i*.*e*., ASV0007, which shared >99% identity with *Clostridium disporicum* and *Clostridium celatum* isolates, and ASV0011, which shared >99% with uncultured bacteria identified from a wastewater treatment system and cultured isolates belonging to *Romboutsia sedimentorum* species (Fig 4).

### Quantification and community analysis of sulfate-reducing bacteria

Quantitative PCR targeting the *dsrA* gene, which encodes the alpha subunit of the dissimilatory sulfite reductase involved in the conversion of bisulfite to sulfide within the dissimilatory sulfate reduction pathway [47], was performed to assess the amount of SRB in both N and T sites. N sediments possessed a significant (p < 0.01) lower number of *dsrA* gene copies per gram compared to T sediments (1.8 x 10^6^ and 3.5 x 10^7^, respectively) (S3 Fig).

**Fig 5:**
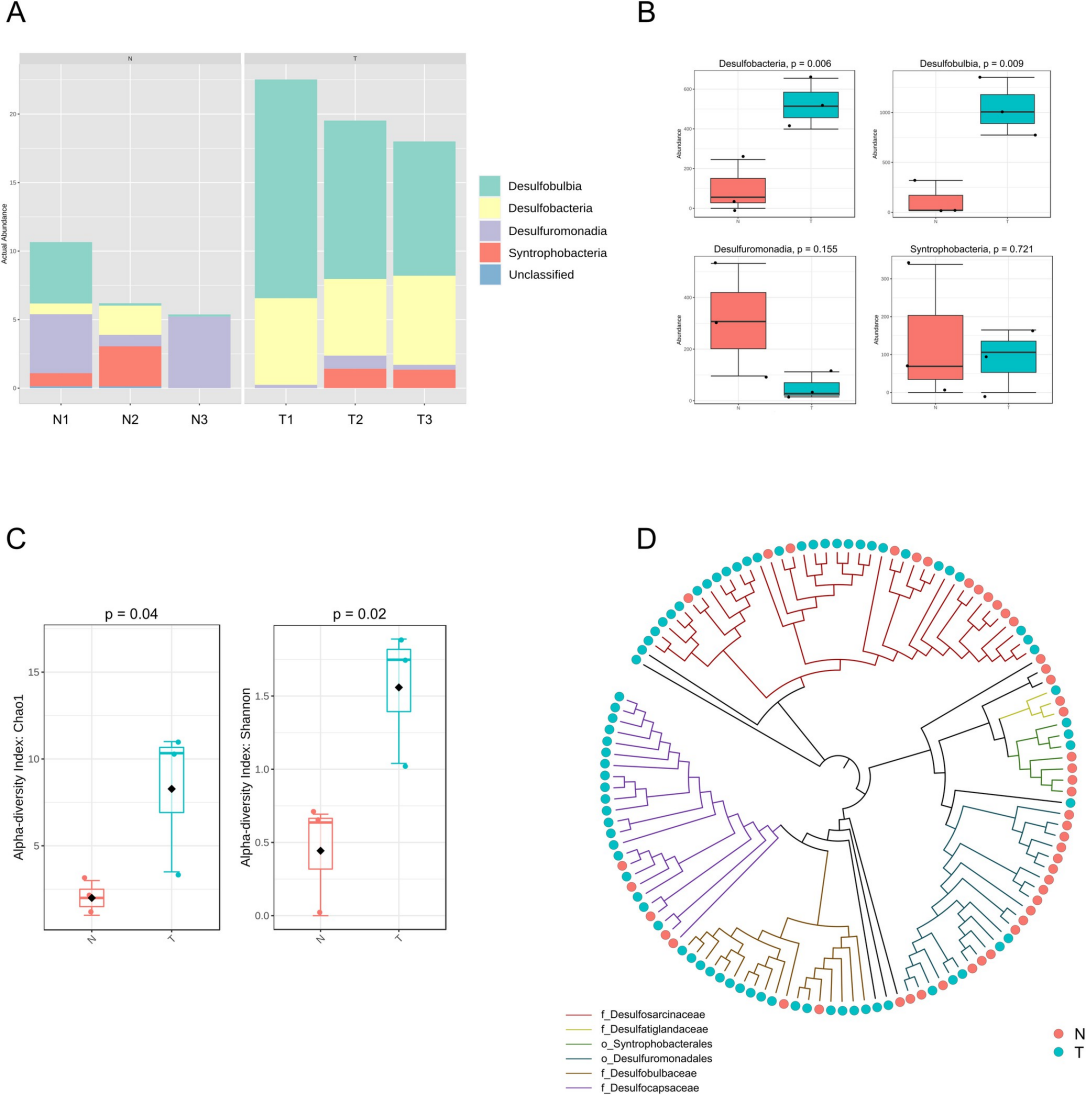
Diversity, taxonomy composition, and phylogenetic analysis of SRB community. A) Taxonomy analysis of *Desulfobacterota* communities at class level. B) Rank test (Anova) of the *Desulfobacterota* classes shown in panel A. C) Chao1 and Shannon diversity indices of *Desulfobacterota* communities in N and T. D) Phylogenetic tree showing all the ASVs affiliated to *Desulfobacterota* from N and T. The color of branches corresponds to orders (preceded by o_ prefix) or families (preceded by f_ prefix) reported in the legend.

To identify possible significant differences among SRB between N and T, further computational analyses of the microbial communities belonging to those bacterial groups known to be potentially involved in the sulfate reduction processes were carried out (Fig 5). SRB are taxonomically affiliated to specific classes within the *Desulfobacterota*, *Firmicutes*, *Nitrospirota*, and *Thermodesulfobiota* phyla [16, 48, 49]. Our sequencing data revealed that in both N and T sediments all the SRB-associated ASVs belonged to the *Desulfobacterota* phylum. *Desulfobacterota* has been proposed as new phylum in 2020 containing the former *Deltaproteobacteria* class and *Thermodesulfobacterota* phlyum [48]. By analyzing the alpha diversity indices at ASV level affiliated to *Desulfobacterota*, richness (Chao1) and diversity (Shannon) were both significantly higher in T compared to N (Fig 5C). T sediments were significantly enriched with *Desulfobulbia* and *Desulfobacteria* classes, which were found in low abundance in N sediments (Fig 5A and 5B). On the other hand, *Desulfuromonadia* and *Syntrophobacteria* classes were slightly more abundant in N compared to T, although without statistical significance. The ASVs belonging to the *Desulfobacterota* phylum were used to build a phylogenetic tree that showed a clear clustering of T-enriching ASVs, which were mainly affiliated with the *Desulfocapsaceae*, *Desulfobulbaceae*, and *Desulfosarcinaceae* families, whereas the N-enriching ASVs were mainly affiliated with the *Desulfuromonadales* and *Syntrophobacterales* orders (Fig 5D). Differently from N-enriching ASVs, most of the ASVs characterizing T sediments were classified up to the genus level and were mainly affiliated to *Desulfobulbus* and *Desulfosarcina*. The *Desulfuromonadia*-affiliated ASVs in N belonged mainly to the Sva1033 family and *Geopsychrobacter* genus (data not shown), whereas *Syntrophobacteria*-affiliated ASVs were all classified up to the *Syntrophobacterales* order.

## Discussion

MIC constitutes a concerning issue for marine infrastructures, especially the long-standing ones, such as wharves or even oil and gas pipelines, since the continuous exposure to seawater microflora leads to corrosion and is responsible for heavy damages [18, 50–52]. Currently employed strategies to prevent MIC in marine infrastructures lack comprehensive microbiological analyses of the installation sites. Additionally, no monitoring procedures aiming at studying the evolution of the microbial communities are usually performed on the sediment sites in the nearby of already installed and operative infrastructures. To get further insights of the microbial communities associated with differentially-impacted marine sites, we analysed the microbial community composition and assessed the SRB abundance in marine sediments collected from nearshore Trieste and offshore Norwegian sites.

The presence of high levels of contaminants, low macrozoobenthic diversity, high organic matter concentration, and high numbers of hydrocarbon-degrading bacteria was reported in previous studies on water and sediments collected from the Trieste port [30, 53]. Indeed, this area possesses contaminants that exceed the legal limits fixed by the Italian government, including mercury, vanadium, zinc, total hydrocarbons, and polycyclic aromatic hydrocarbons [30–32]. By combining the experimental data on the collected sediments either performed in our laboratory or by Saipem S.p.A. sediments collected from the Trieste port area showed significantly higher concentration of total organic carbon and total hydrocarbons in T compared to N (S2 Table), indicating both a higher anthropic contamination and higher proneness to possible MIC processes in the Trieste port area. We intended to focus the attention on the marine communities inhabiting these two sites and their impact on the associated MIC hazard. Therefore, we opted not to take into account the features, characterizing the marine infrastructures (*i*.*e*., their materials and coatings), which also contribute to determine the entity of MIC events.

The sediments collected at different distances of N and T sites presented similar microbial community structures among the three samples of each group, that significantly differed between the two sites. However, no significant differences were observed in terms of microbial richness and diversity between N and T. This indicates that the environmental settings of the two sites shape the taxonomy composition of the global microbial communities without affecting their richness and diversity. Conversely, the main bacterial group distribution varied greatly between N and T. Many of the differently-distributed bacterial taxa can be considered bioindicators of the health conditions of the marine sediments under analysis. Among the most abundant phyla, *Bacteroidota* and *Firmicutes* were associated with T sediments and their members suggested high human activity compared to N sediments. This was highlighted by the significantly enriched genera and most abundant ASVs in T affiliated to anaerobe species belonging to the *Clostridium* and *Rombutsia*, which are indicator of sewage solids, and which are similar to clones previously associated to wastewater treatments, anaerobic digesters, and fecal contamination [54, 55]. Trieste sediments were also characterized by the methanotrophic *Methyloceanibacter* genus, previously associated with marine sediments in the proximity of hydrothermal vents [56, 57]. Although this bacterial genus is commonly associated with hydrothermal vents, its enrichment in T can be associated with anthropogenic contamination from synthetic fertilizers that are poured into the sea [58].

Differently from T, N sediments were significantly enriched in some bacterial groups that are associated with pristine/healthy environments and poorly impacted by human contamination. In particular, N was enriched in *Nitrospinota* and *Nitrospirota* phyla and in *Nitrospina* and *Nitrospira* genera, whose members are the main responsible for the nitrification processes in marine environments by performing oxidative reaction of nitrite to nitrate [59, 60]. Moreover, these genera provide pioneering metabolic activities for the establishment of the entire microbial community and for the maintenance of the global nitrogen cycling [61]. N sediments were also significantly enriched in *Woeseia* genus, whose members are commonly found in marine sediments communities and are characterized by a facultative chemolithoautotrophic metabolism [62, 63]. Their contribution in the inorganic and organic carbon cycling is hypothesized to be crucial for the marine ecology in pristine seafloor sediments and the abundance of these microbial members in N sediments represents an additional indication of the ecological health of the offshore sampling site. Despite the most abundant bacterial taxa retrieved from N sediments suggest a low impact of contaminants, the phylogenetic analysis of the most abundant ASVs in N sediments indicated nucleotide similarities with clones identified from the oil-contaminated marine site in the Gulf of Mexico after the Deep Horizon platform explosion in 2010 [45, 46]. These ASVs were mostly unclassified at order, family, or genus level, and were affiliated to *Alphaproteobacteria*, *Methylomirabilota*, and *Actinobacteriota*. Although similar environmental damages have never been documented in the Barents Sea sampling site, this finding suggests that the presence of the drilling platforms close to the collected sediments might contribute to the selection of these specific microbial group, which are still poorly characterized and/or have not been isolated yet.

One of the main issues of the contamination of marine sediments concerns the possible enrichment of microorganisms that can be harmful for marine pipeline systems [19, 51]. The installation of infrastructures in marine environments needs to consider the microbial community inhabiting the putative dedicated location, with the aim of preventing/retarding the establishment of MIC processes [18]. Although the main species responsible for MIC are positively selected by the formation of thick biofilms on the metal surface of infrastructures, these microorganisms are endemic and already present in the anoxic niches of the bent sediments [51]. Moreover, model studies have demonstrated that the microorganisms from marine sediments enhanced both biofouling and biocorrosion in model tank reactors [64]. Indeed, MIC management is mainly reactive rather than being proactive, including the use of physical treatments as pigging and ultraviolet irradiation, as well as broadband biocides endowed with high toxicity [65]. Very few studies have considered the evolution of the microbial community during the usage of an infrastructure over time, and no specific microbiological investigations of marine water and sediments are usually planned before the installation of marine infrastructures. Our data reveled a highly diversified microbial community between the two sites, with T sediments harboring a large number of bacteria involved in MIC, suggesting a higher susceptibility of port and harbors’ infrastructures to corrosion [66]. We performed deeper analyses targeting the portion of microbial communities potentially involved H_2_S production, which revealed a significant enrichment in T compared to N in terms of both relative abundance of SRB (through 16S rRNA-targeting Illumina sequencing) and total number of SRB cells quantification (through *drsA*-targeting qPCR). These data confirm that T sediments harbor a significant higher number of SRB cells compared to N sediments. Additionally, the SRB communities of the two sites were taxonomically different. T sediments were enriched in SRB species belonging to the *Desulfobulbaceae* and *Desulfosarcinaceae* families, *i*.*e*., *Desulfobulbus* and *Desulfosarcina* genera. Both these genera live in anoxic environments, they are highly abundant in sediments with high concentration of organic compounds where they perform organic matter mineralization [67–69]. *Desulfosarcina* belongs to the *Desulfosarcina*/*Desulfococcus* (DSS) clade, which is considered one of the most important lineages involved in hydrocarbon degradation within marine sediments [70]. Additional SRB significantly enriched in T belonged to the Sva0485 clade, which is a marine benthic group (MBG) widely distributed in marine sediments playing a key role in carbon cycling in organic-rich marine environments and representing an important sink of acetate and hydrogen in coastal marine sediments [71–73]. If on one hand the sulfate reducing *Desulfobulbus* and *Desulfosarcina* were highly present in T and associated with high amount of organic matter in the marine sediments, the *Desulfobacterota* community in N was characterized by other SRB members that are not typically correlated with locations featured by pollution/organic compounds, and that contribute to the sulfur cycle in the marine environment. Among these, the Sva1033 family and the *Geopsychrobacter* genus were the most abundant taxa identified in N. *Geopsychrobacter* and Sva1033 members are psychrotolerant and psychrophile, respectively, are frequently associated with Antarctic and Arctic marine sediments, and possess iron-reducing capacities contributing to the sulfur metabolism [74–76]. We are well aware that characterizing the SRB population abundance and composition is not sufficient to reliably infer the proneness of a specific site to be affected by MIC, as many other microbial phyla could contribute to this process [77, 78]. However, these data might indicate that the detection of specific microbial classes could predict the corrosion rate characterizing a defined environment, especially when suffering from anthropic impact. This seems to be the case of the T site, which also resulted enriched of bacterial groups capable of contributing to the production of corrosive sulfur species. Chemolithoautotrophic sulfur-oxidizing bacteria belonging to the *Thiogranum* genus were significantly present in T compared to N sediments, whose members were isolated from marine environments and are capable of oxidizing reduced sulfur compounds [79, 80]. Their presence in T is likely attributable to the low depth of the sampling site, in which oxygen is more available compared to the deeper sampling sites in N.

## Conclusions

MIC is one of the main consequences associated with anthropic contamination of marine environments and is dependent on the establishment of specialized SRB. The investigation of the microbial communities inhabiting marine sediments is a key factor in the identification of healthy and suitable locations for the establishment of infrastructures. Further, the monitoring of the microbial community evolution in the proximity of in-use marine infrastructures should be promoted to prevent and/or mitigate MIC events. The enrichment of human-associated and sulfate-reducing bacterial members in Trieste demonstrated that this site is featured with high anthropogenic impact and is more likely to host corrosion events on installed marine infrastructures than the Norwegian site. On the other hand, some SRB members were also detected in the Norwegian sediments, suggesting that periodic monitoring of the evolution of the microbial communities at this location should be promoted and regulated to mitigate possible MIC processes. With the recent advancement of omic sequencing technologies, these analyses are now economically accessible and allow reliable results to be obtained in a very short time. Implementation of the survey already in use with microbial community studies would provide a comprehensive scenario of potential corrosion processes and represents a proactive complementary strategy to detect MIC at an early stage and to mitigate MIC already underway.

## Supporting information

S1 Table Geographical characteristics of Norway (N1-3) and Trieste (T1-3) sediments analysed in this work and sequencing results. (DOCX)

S2 Table Environmental parameters from N and T sample sites. TOC = Total Organic Carbon; THC = Total Hydrocarbon Concentration; PAH = Polycyclic Aromatic Hydrocarbons.
(DOCX)

S1 Fig Rarefaction curves. Rarefaction curves of the microbial communities in offshore Norway (N) and nearshore Trieste (T) samples, showing the number of Amplicon Sequence Variants (ASVs, y axis) against the number of obtained reads (x axis).
(TIF)

S2 Fig Most abundant microbial lineages. Microbial community composition of the offshore N and nearshore T sediments at the lowest taxonomy classification with relative abundances >3% in at least one sample. The gray scale color indicates the abundance of each taxa.
(TIF)

S3 Fig Abundance of sulfate-reducing bacteria. Quantitative PCR targeting the *dsrA* gene from the offshore N and nearshore T sediments.
(TIF)
