# Supplementary material for: Characterization of bacterial communities associated with seabed sediments in offshore and nearshore sites to improve Microbiologically Influenced Corrosion mitigation on marine infrastructures
Source: PLoS One. 2024 Sep 4;19(9):e0309971. doi: 10.1371/journal.pone.0309971 (PMC11373832; doi:10.1371/journal.pone.0309971)
Supplement: S1 Table — (DOCX) [file pone.0309971.s001.docx]

**S1 Table.** Geographical characteristics of Norway (N1-3) and Trieste (T1-3) sediments analysed in this work and sequencing results.

| **Sediment** | **Location** | **Coordinates** | **no. reads** | **no. ASVs** |
| --- | --- | --- | --- | --- |
| N1 | Norway | 22°18.4999’E  71°18.2653’N | 7,141 | 152 |
| N2 | Norway | 22°18.5082’E  71°18.2669’N | 11,530 | 489 |
| N3 | Norway | 22°18.5001’E  71°18.259’N | 10,194 | 195 |
| T1 | Trieste | 13°45.6361’E  45°39.8362’N | 6,305 | 155 |
| T2 | Trieste | 13°45.6646’E  45°39.8404’N | 11,702 | 471 |
| T3 | Trieste | 13°45.6766’E  45°39.8438’N | 7,899 | 158 |
