# Supplementary material for: Characterization of bacterial communities associated with seabed sediments in offshore and nearshore sites to improve Microbiologically Influenced Corrosion mitigation on marine infrastructures
Source: PLoS One. 2024 Sep 4;19(9):e0309971. doi: 10.1371/journal.pone.0309971 (PMC11373832; doi:10.1371/journal.pone.0309971)
Supplement: S2 Table — TOC = Total Organic Carbon; THC = Total Hydrocarbon Concentration; PAH = Polycyclic Aromatic Hydrocarbons. (DOCX) [file pone.0309971.s002.docx]

**S2 Table. Environmental parameters from N and T sampling area.** TOC = Total Organic Carbon; THC = Total Hydrocarbon Concentration; PAH = Polycyclic Aromatic Hydrocarbons.

| **Site** | **pH**^a^ | **Temperature^b^**  **(°C)** | **Salinity^c^**  **(g/kg)** | **TOC**  **(%)** | **THC**  **(mg/kg)** | **PAH**  **(µg/kg)** |
| --- | --- | --- | --- | --- | --- | --- |
| **N** | 7.08 ± 0.2 | 4.2 ± 0.4  (3.9) | 35.1 ± 0.0 | < 1^d^ | 5.03 ± 0.8^d^ | 211 ± 4^d^ |
| **T** | 7.28 ± 0.2 | 15.3 ± 5.7  (6.9) | 36.7 ± 0.9 | 2.30 ± 0.4^e^ | 151 ± 70.3^e^ | > 500^f^ |

**^a^** Data experimentally measured in lab, upon samples reception.

**^b^** Values represent the annual seawater temperature at the sampled depth (*i.e.,* 400 mt for N and 10 mt for T), the values in parentheses are indicative of the month of March in which samples were collected. These data were extracted from the NOAA World Ocean Atlas 13 (WOA13) database (https://www.nodc.noaa.gov/OC5/woa13/woa13data.html), and provided by Saipem S.p.A.

**^c^** Values represent the annual seawater salinity at the sampled depth (*i.e.,* 400 mt for N and 10 mt for T). These data were extracted from the NOAA World Ocean Atlas 13 (WOA13) database (https://www.nodc.noaa.gov/OC5/woa13/woa13data.html), and provided by Saipem S.p.A.

**^d^** Data for the N site were retrieved from mareano.no database, mediating values from sites in the nearby coordinates.

^e^ Data for the T site were collected by the Istituto Nazionale di Oceanografia e di Geofisica Sperimentale (https://www.ogs.it) and provided by Saipem S.p.A. Analyzes were conducted in triplicate by Accredia accredited laboratory according to ISO 17025:2005 standard for all the analytes researched and operating with a quality system certified according to ISO 9001:2015 standard.

**^f^** PAH value was retrieved from literature [30-32].
