# Supplementary figures and images for: Characterization of bacterial communities associated with seabed sediments in offshore and nearshore sites to improve Microbiologically Influenced Corrosion mitigation on marine infrastructures
Source: PLoS One. 2024 Sep 4;19(9):e0309971. doi: 10.1371/journal.pone.0309971 (PMC11373832; doi:10.1371/journal.pone.0309971)

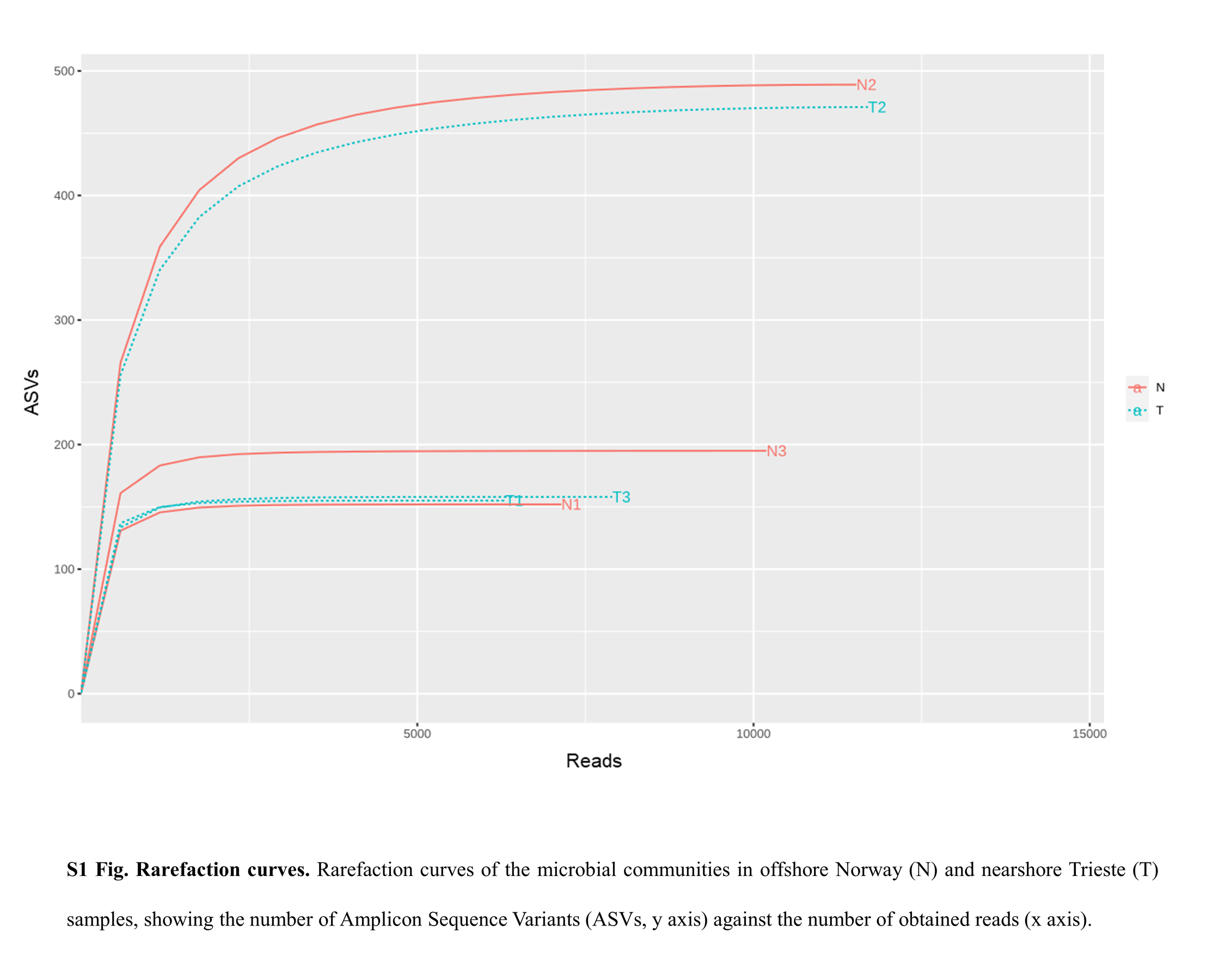

Supplement: S1 Fig — Rarefaction curves of the microbial communities in offshore Norway (N) and nearshore Trieste (T) samples, showing the number of Amplicon Sequence Variants (ASVs, y axis) against the number of obtained reads (x axis). (TIF) [file pone.0309971.s003.tif]

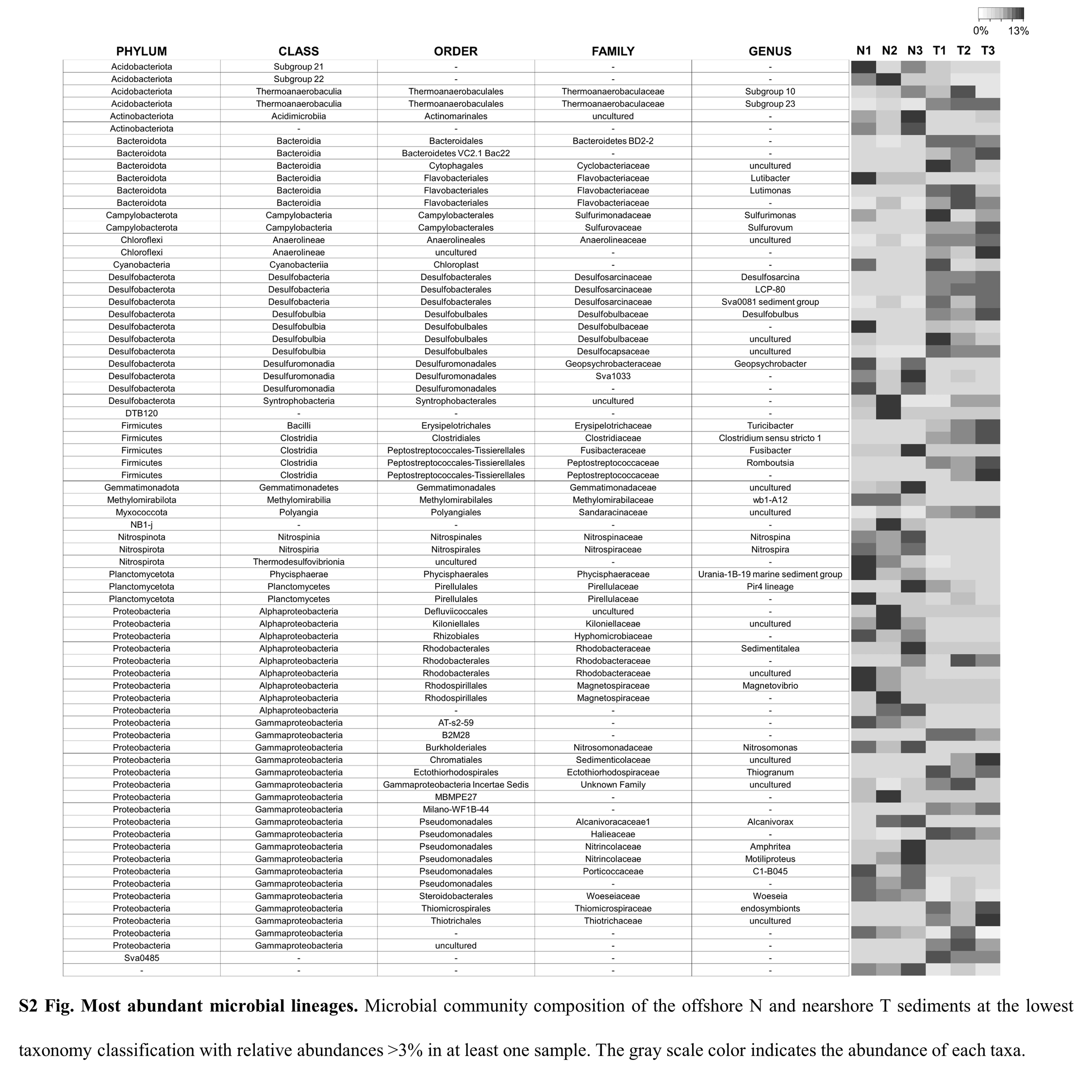

Supplement: S2 Fig — Microbial community composition of the offshore N and nearshore T sediments at the lowest taxonomy classification with relative abundances >3% in at least one sample. The gray scale color indicates the abundance of each taxa. (TIF) [file pone.0309971.s004.tif]

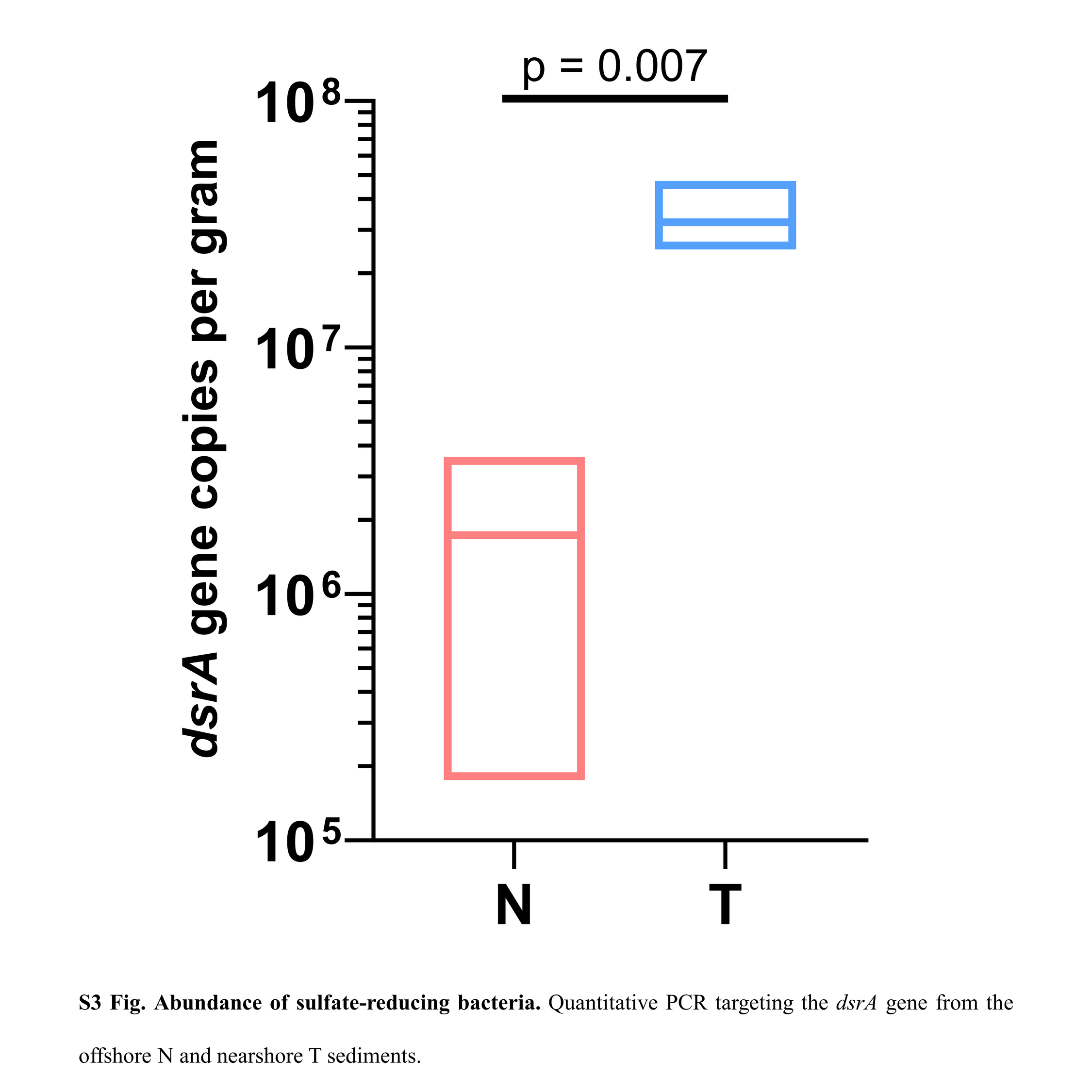

Supplement: S3 Fig — Quantitative PCR targeting the dsrA gene from the offshore N and nearshore T sediments. (TIF) [file pone.0309971.s005.tif]
